# Case Report: From Kaposi’s sarcoma to primary effusive lymphoma

**DOI:** 10.3389/fmed.2025.1591462

**Published:** 2025-08-12

**Authors:** Tao Wang, Changsheng Xia, Yanni Deng, Liangbi Xu, Yanping Jiang

**Affiliations:** ^1^Department of Dermatology, The Affiliated Hospital of Guizhou Medical University, Guiyang, China; ^2^Digestive Endoscopy Center, The Affiliated Hospital of Guizhou Medical University, Guiyang, China

**Keywords:** human herpesvirus 8, lymphoma, Kaposi’s sarcoma, Epstein–Barr virus infections, ethnicity

## Abstract

Primary effusion lymphoma (PEL) is a rare B-cell lymphoma with an extremely poor prognosis that is associated with long-term persistent latent infection with Kaposi’s sarcoma-associated herpesvirus (KSHV) and Epstein–Barr virus (EBV). However, studies on the correlation between KSHV genotype and PEL development in elderly patients are still lacking. We present the first global case of non-HIV, non-effusive, difficult-to-diagnose PEL with disseminated Kaposi’s sarcoma (KS) in an elderly patient of Bouyei nationality, dynamically demonstrating that KSHV and EBV co-infection promote tumorigenesis. Phylogenetic analysis based on the Open Coding Framework (ORF)-K1 gene indicated that five samples from the patient’s blood (mtl A), saliva (mtl B), descending colon (mtl C), skin (mtl D), and gastric mucosa (mtl E) may belong to a new subtype, Cnew. KSHV genotypes appear to show a pattern of traceability consistent with the human Y-chromosome DNA haplogroup tree.

## 1 Introduction

Co-infection is of particular human health importance because pathogens can interact within the host and have synergistic effects on transmission and disease progression. Moreover, co-infection promotes the occurrence and development of malignant tumors, especially the co-infection of viruses, which induces cancers accounting for 15–20% of human cancers worldwide (1–3). The most representative are Kaposi Sarcoma-associated herpes virus (KSHV), also known as human herpesvirus-8 (HHV-8) and Epstein–Barr virus (EBV), which are the only two human carcinogenic *γ*-herpesviruses currently known, each accounting for 1–2% of all 20% of the infectious disease-associated malignancy burden in humans. Together, they act on B cells, contributing to the formation of specific primary effusion lymphoma (PEL) (4–6).

It is defined as a rare, aggressive B-cell non-Hodgkin lymphoma usually presenting as serous effusion (7), and PEL indeed represents a natural experiment in which two viruses, EBV and KSHV, are stably co-infected in most tumor cells (8). Although human immunodeficiency virus (HIV) is also involved (9), it is generally understood as one of the immunodeficiency states of PEL lymphomagenesis, other established risk factors include iatrogenic immunodeficiency after solid organ transplantation, cirrhosis, cancer, aging or immunosenescence, and other immunosuppressive conditions (9). Among them, immunosenescence may be the main cause of PEL in non-HIV patients, such as diffuse large B-cell (DLBCL), which is more common in the elderly (10). One of the key factors linking aging to lymphomagenesis is that it promotes chronic infections and leads to defective antitumor immunity (9).

Therefore, in the aging societies, unraveling the process by which EBV and KSHV induce B cells to develop into lymphoma in immunosenescent hosts is of great significance not only for the health of the elderly, but also for advancing tumor therapeutics. Unfortunately, until now, there has been a lack of in-depth and complete clinical and basic studies on natural aging HIV-negative PEL cases worldwide (9, 11). It is also unclear whether the six known KSHV (ORF)-K1 genotypes (A-F) and numerous subtypes are related to the occurrence, progression, clinical phenotype and prognosis of PEL (12, 13).

Here, we report a case of KSHV and EBV co-infection in a non-HIV, non-effusive, diagnostically-challenging elderly male PEL with disseminated KS to raise awareness of this rare malignant lymphoma spectrum disease. To the best of our knowledge, this case is the first report of KSHV-associated malignancy in the Bouyei population worldwide, and this interesting point prompted us to complete a phylogenetic analysis to further explore the correlation between genotype and clinical manifestations.

## 2 Case presentation

An 83-year-old male of Bouyei nationality in Sandu, South Guizhou Province was admitted to the Department of Dermatology of our hospital in January 2018 presenting with cutaneous rash involving limbs and fever. The initial symptom was a painless and itchy purplish-red rash on the back of both feet, which had been present for 4 years. Two years later, the rash gradually spread to the trunk and limbs, accompanied by swollen lymph nodes in the neck and both groin, intermittent fever and chills, especially at night, with a body temperature up to 39°C. The rash worsened with fever and was accompanied by mild itching. In the past year, the patient’s symptoms worsened and his body weight decreased by 15 kg. He had a history of hypertension for 2 years, no history of infection, and no special family history.

**Figure 1:**
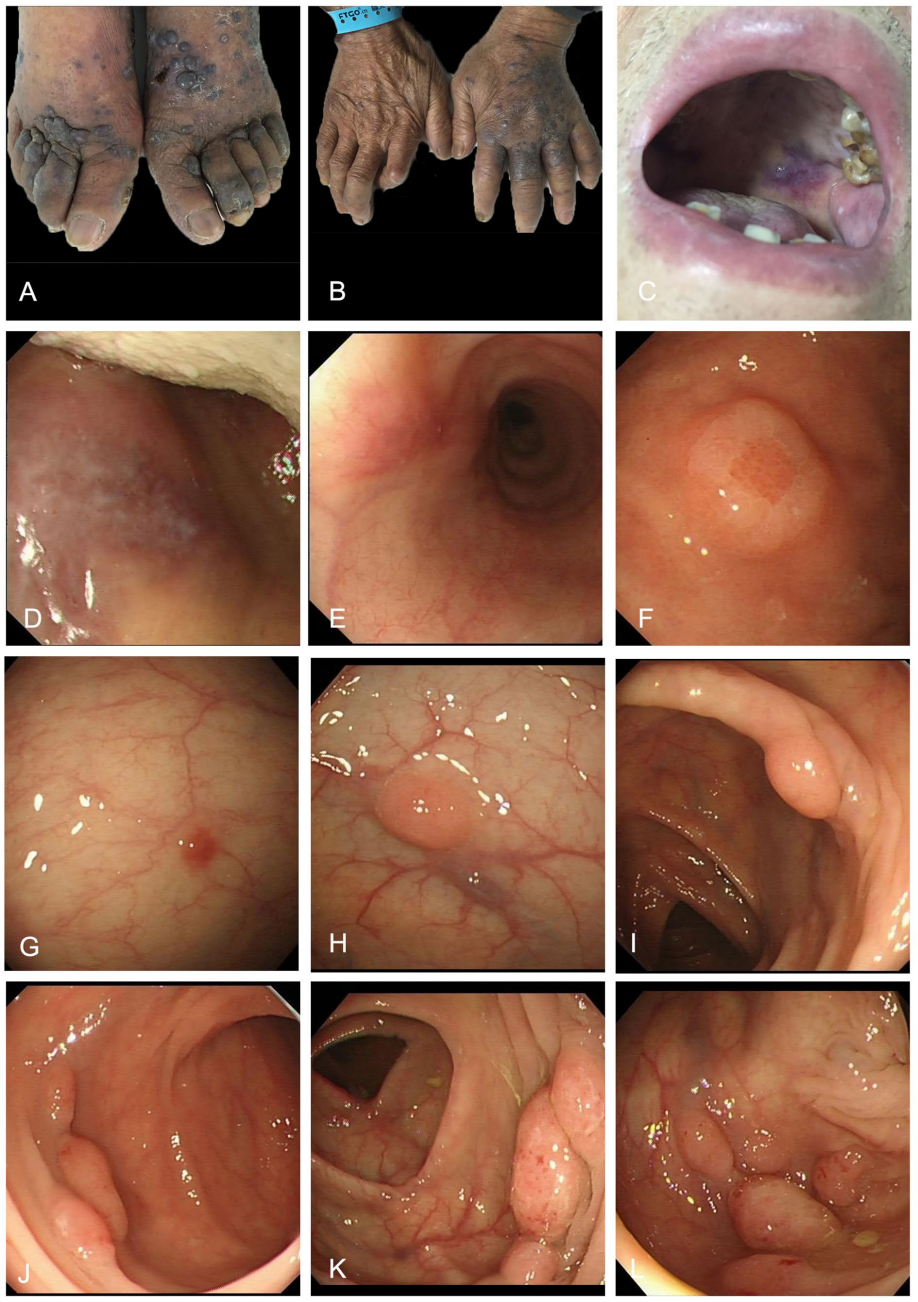
Purple plaques on the dorsum of feet **(A)** and hands **(B)** and oral mucosa **(C)**. Endoscopic images of the gastrointestinal tract. Gastroscopy showed oral erythema **(D)** and multiple polypoid protrusions in the esophagus **(E)**, gastric fundus and gastric body **(F)**. Colonoscopy showed a single polypoid mass in the rectum, sigmoid colon and hepatic flexure of colon **(G–I)**; Colonoscopy revealed multiple pink polypoid masses in the descending colon **(J–L)**.

Physical examination revealed multiple lymph nodes (0.5–1.5 cm diameter) that were palpable in the left anterior neck and both inguinal regions. These lymph nodes were firm, nonadherent, and exhibited good mobility. There was purple nodules and plaques (1–2 cm diameter) scattered on the trunk and limbs, partially fused, especially on the dorsum of feet and hands (Figures 1A,B). A purplish erythematous macule (about 2 cm diameter) was seen in the mucosa of the palate without surface ulceration (Figure 1C). Endoscopy of the gastrointestinal tract showed oral erythema (Figure 1D) and multiple polypoid protrusions in the esophagus (Figure 1E), gastric fundus and gastric body (Figure 1F). Colonoscopy showed a single polypoid mass in the rectum, sigmoid colon and hepatic flexure of colon (Figures 1G–I), but the polypoid mass in the descending colon increased significantly (Figures 1J–L), with a diameter of 0.7–1.5 cm, clear boundaries and flexible texture.

Routine blood test showed that RBC decreased to 3.14 g/L (normal range 4.30–5.30 g/L) and hemoglobin (Hb) decreased to 90.00 g/L (normal range 130.00–175.00 g/L). Liver and kidney function tests showed that albumin decreased to 22 g/L (normal range 40–55 g/L). C-reactive protein (CRP) was elevated to 76 mg/L (normal range 0–8 mg/L); Negative for hepatitis B and HIV; Antinuclear antibodies (ANA) were negative; *Mycobacterium tuberculosis* specific cellular immune antibody test (QFT) was negative; Immunofixation electrophoresis showed elevated gamma globulin, mainly *κ* light chain IgG. The level of κ light chain in peripheral blood and urine was 1870.00 mg/dL (629.00–1350.00 mg/dL) and 16.50 mg/dL (0.00–1.85 mg/dL), respectively. The *λ* light chain values in blood and urine were normal. Ferritin increased to 600 ng/mL (0–322 ng/mL); Anti-EB virus capsid antibody (IgG) and high affinity antibody (IgG) were positive; The complete set of virus antibody (TORCH) was positive for CMV-IgG. Routine bone marrow examination showed hyperplastic anemia, increased plasma cells, and hemophagocytosis. The absolute lymphocyte count showed that CD4/CD8 ratio decreased to 0.74 (1.04–1.72), and the absolute count and percentage of CD4 and NK cells decreased, with the former absolute count decreasing to 218 cells/ul (410–1,590 cells/ul). Routine urine and stool tests were normal. Bone scan was normal. Chest CT and abdominal ultrasound were normal.

**Figure 2:**
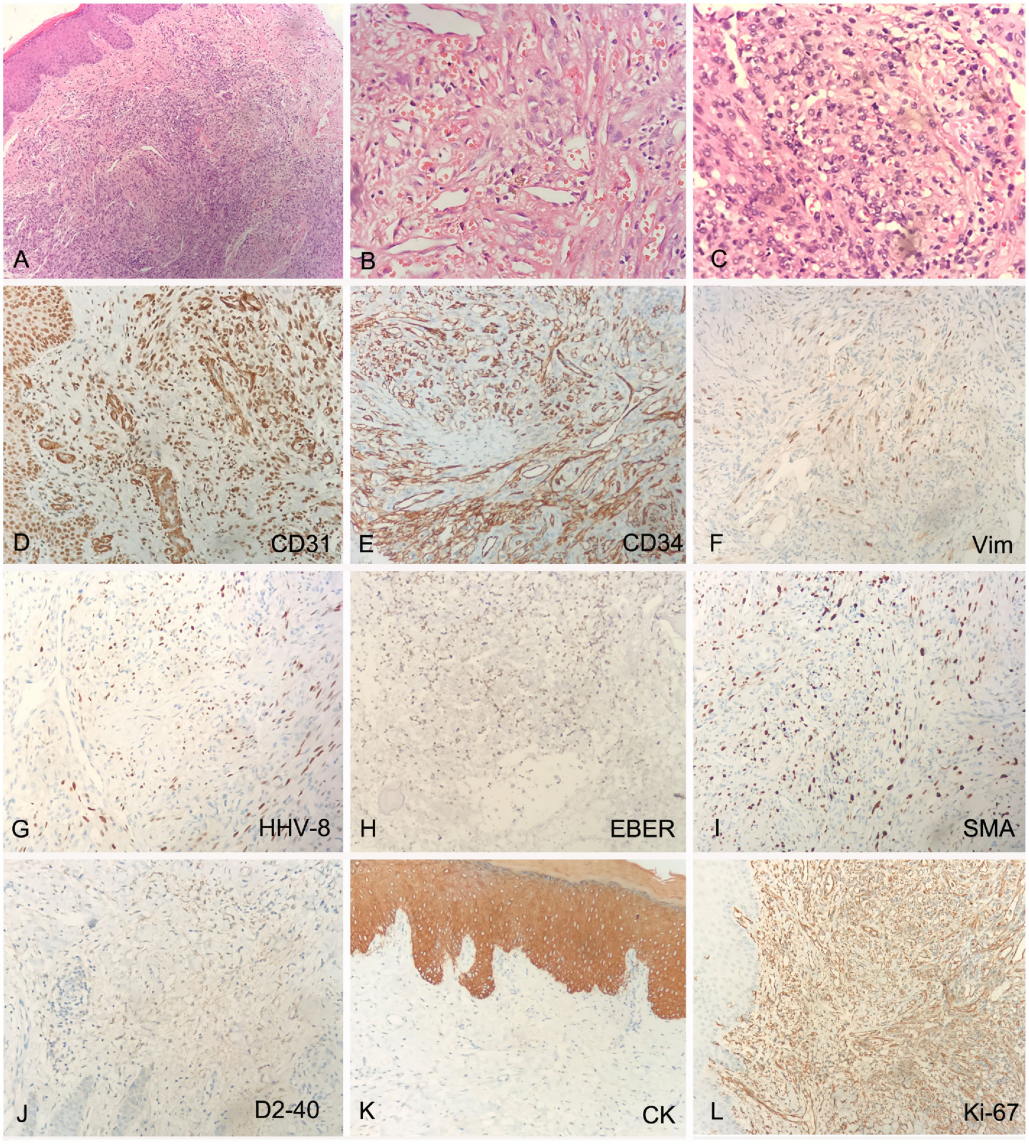
Histology and IHC of the mass in the right foot. The perivascular spindled cells with extravasated erythrocytes and hemosiderin at lower (**A**, H&E, original magnification 20x) and higher (**B,C**, H&E, original magnification 40x) magnification; The spindle cells were positive for CD31, CD34, Vimentin (Vim), HHV-8, EBV-encoded RNA in-situ hybridization (EBER-ISH) and smooth muscle actin (SMA) (**D–I**, original magnification 20x), negative for D2-40 and CK (**J,K**, original magnification 20x), Ki-67 proliferation index was up to 30% (**L**, original magnification 20x).

**Figure 3:**
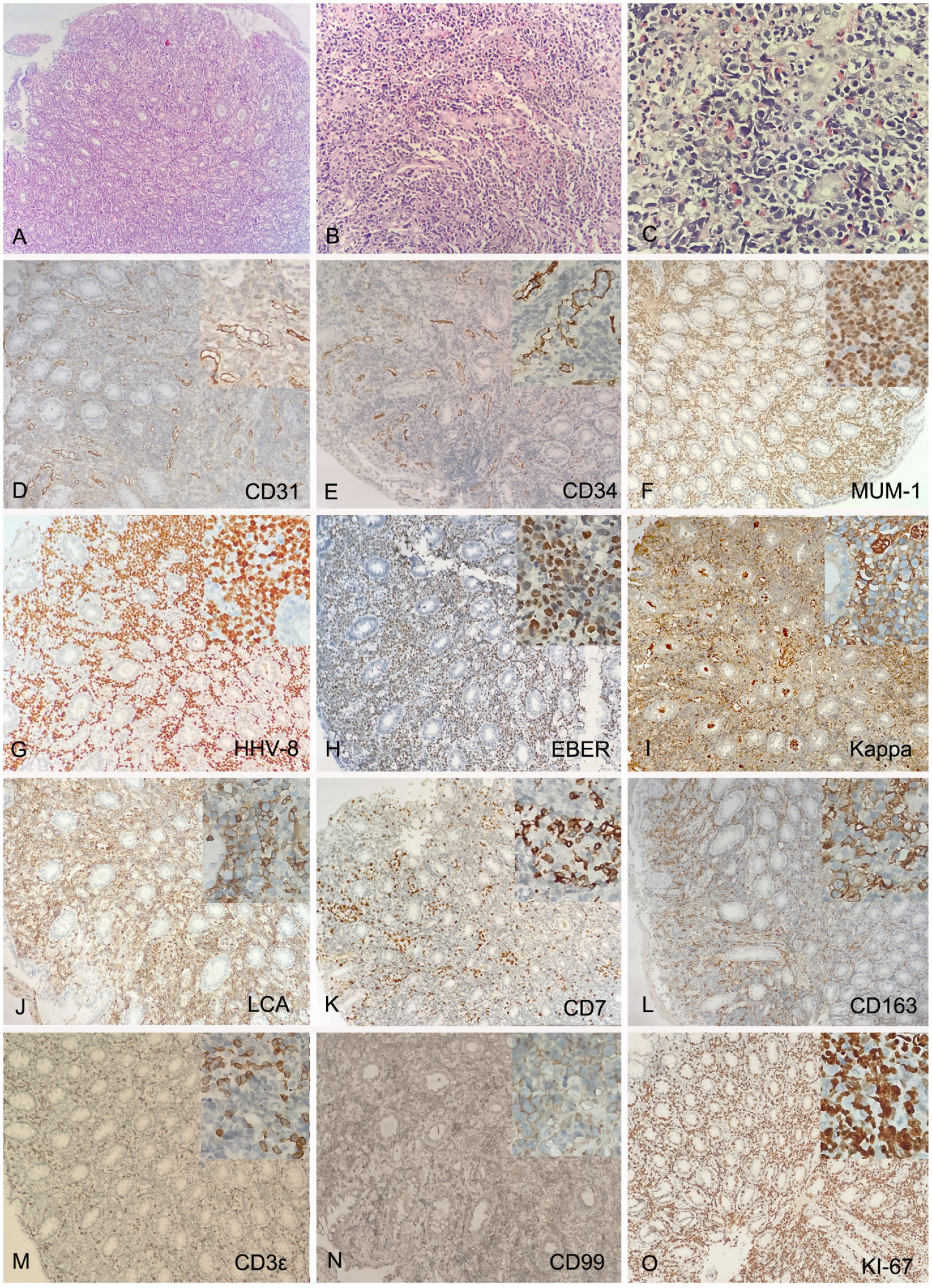
Histology and IHC of the mass in the descending colon. Biopsy showed diffuse proliferation of large lymphoma cell in the lamina propria of mucosa at lower (**A**, H&E, original magnification 10x) and higher (**C**, H&E, original magnification 40x) magnification, co-existent with a proliferation of slit-like vessels, filled with red blood cells surrounded by slightly atypical spindle cells (**B**, H&E, original magnification 20x). The vascular lesion showed expression of CD31 and CD34 in the few spindle cells (**D,E**, original magnification 20x); The lymphoma cells were positive for IRF4/MUM1, HHV-8, EBER, monotypic kappa and CD45/LCA (**F–J**, original magnification 20x), and partially positive for CD7, CD163, CD3ε, and CD99 (**K–N**, original magnification 20x), negative for CD79a, CD20, Pax-5, Bcl-2 and C-myc. The Ki-67 proliferation index was greater than 95% (**O**, original magnification 20x). Inset represent magnification 40x.

The pathological biopsy of the left foot skin tissue suggested proliferation of fusiform cells and endothelial cells, with extravasation of red blood cells and intervening slit-like spaces (Figures 2A–C). Immunohistochemical analysis (IHC) showed that the spindle cells were positive for CD31, CD34, Vimentin (Vim), HHV-8, EBV-encoded RNA in-situ hybridization (EBER-ISH) and smooth muscle actin (SMA), but negative for D2-40, CK and HMB45. The Ki-67 proliferation index was found to be up to 30% (Figures 2D–L). Multiple biopsies of the antrum, corpus, ileum and rectum showed chronic inflammation, with intervening slit-like spaces and extravasation of red blood cells (Supplementary Figures S1A–D). The pathological examination of descending colon biopsy revealed diffuse proliferation of large lymphoma cell in the lamina propria of mucosa, with round nuclei, prominent nucleoli and abundant cytoplasm, co-existent with a proliferation of slit-like vessels, filled with red blood cells surrounded by slightly atypical spindle cells (Figures 3A–C). Immunohistochemically, the vascular lesion showed expression of CD31 and CD34 in the few spindle cells (Figures 3D,E); the lymphoma cells were positive for IRF4/MUM1, HHV-8, EBER, CD45/LCA and cytoplasmic immunoglobulin kappa light chain restriction (Figures 3F–J), and partially positive for CD7, CD163, CD3ε, and CD99 (Figures 3K–N), negative for CD79a, CD20, Pax-5, Bcl-2 and C-myc. The Ki-67 proliferation index was greater than 95% (Figure 3O).

Lesions were observed in both the skin and gastrointestinal tract and were diagnosed as disseminated KS, while the descending colon mass continued to evolve into a highly aggressive B-cell lymphoma. The patient was eventually diagnosed with non-HIV extracavitary primary effusion lymphoma (EC-PEL) with disseminated KS. Due to the patient’s older age and poor basic condition, he refused to be hospitalized for further treatment and passed away 6 months later.

## 3 Phylogenetic analysis

Peripheral blood and saliva samples were collected from the patient, labeled as mtl A and mtl B. Fresh biopsy tissues from the descending colon, foot skin, and gastric antrum masses were also collected and labeled as mtl C, D, and E. We successfully amplified all samples for sequencing of the VR1 region of HHV-8 ORF-K1 sequence (about 370 bp). The sample labeled yyz (JYP 2016) is from our previous work (14). The 6 new nucleotide sequences were all deposited in GenBank under accession numbers OR 253065 to OR 253070.

**Figure 4:**
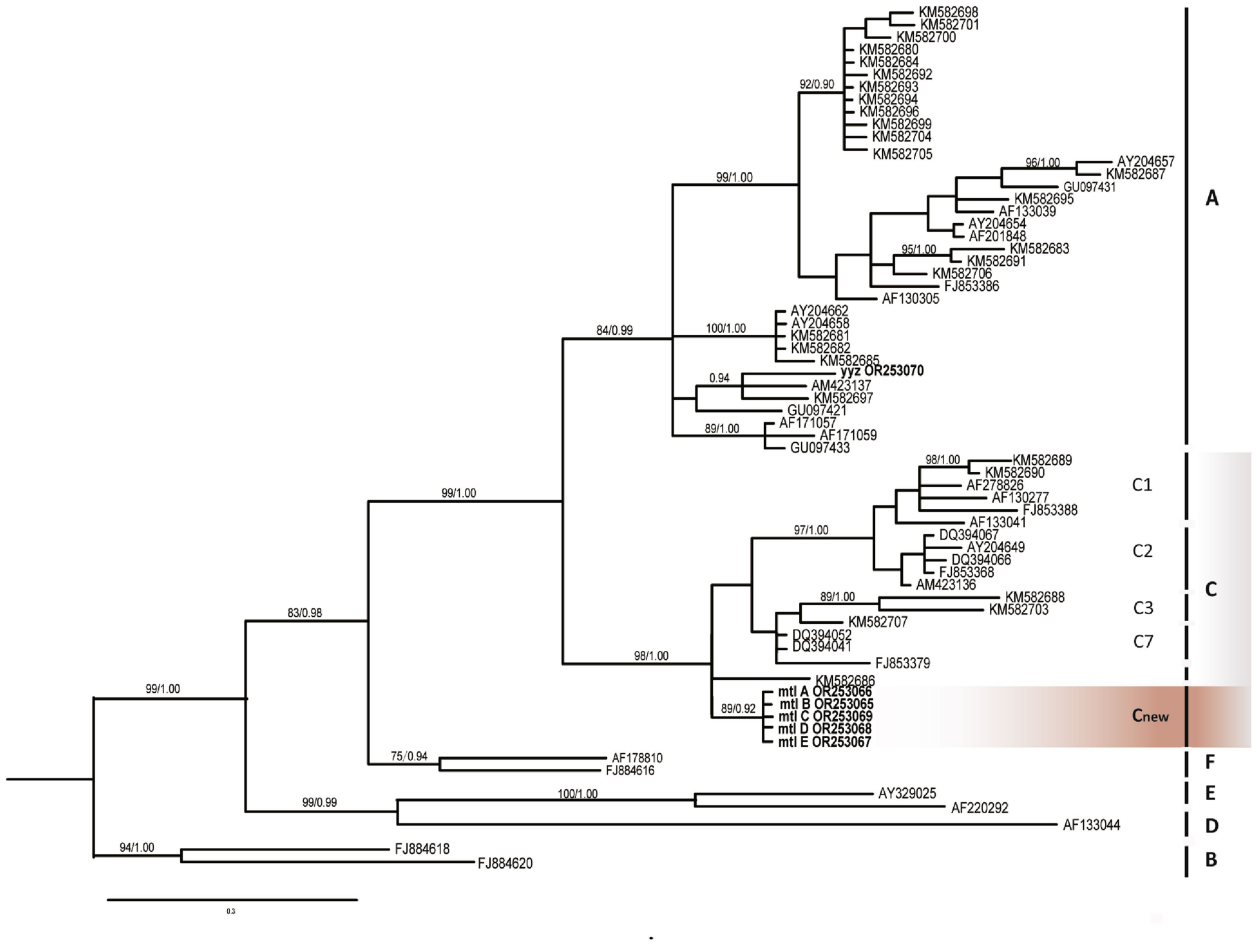
Phylogenetic tree constructed through Bayesian inference analyses based on ORF-K1 sequences showing relationships of seven genotype belonging to human herpesvirus 8 (HHV-8). Only support values exceeding bootstrap values of 70% and Bayesian posterior probabilities of 0.95, respectively, are shown. Bars on the right indicate subtypes, groups. B subtype was used as the outgroup. The new Guizhou sequences (mtl A, B, C, D, E) were all included in the C group (gray) and clustered in an interval different from other C1, C2, C3, and C7 variants, marked as Cnew variant (orange). Guizhou samples are highlighted in bold.

This phylogenetic analysis (Figure 4) clearly distinguishes the six known genotypic clades (A, B, C, D, E, F), and all these branches have high bootstrap values. Our samples can be clearly categorized into two very distinctive groups corresponding to previously assigned A and C subtypes (ML/BI 99/1.00). The sample yyz obtained from the skin tissue of a case of KS of Miao nationality in Guizhou belongs to the subtype A (14), the other five samples from the same patient in this study are all involved in subtype C (ML/BI 98/1.00) and clusters into a single subclade with strong bootstrap support (ML/BI 89/0.92), which is completely different from the previously defined variant subtypes of C1, C2, C3 and C7 (12). We tentatively defined this variant subgenotype as Cnew. Five samples were completely consistent without nucleotide difference.

## 4 Discussion

To date, the diagnosis and treatment of PEL, a rare B-cell lymphoma, remain a challenge, especially in non-HIV, non-effusion cases with clinical manifestations (9, 11). The diagnostic clue in this patient was the typical KS skin lesions, while the single purplish red macule in the oral mucosa and subsequent gastrointestinal endoscopy revealed evidence of gradual progression of KS from the skin to the gastrointestinal tract (GI) (15). Subsequently, the additional diagnosis was made on pathological examination of a solid polypoid mass in the descending colon. Diffuse lymphoma cells in the lamina propria of this site strongly expressed the plasma cell differentiation marker IRF4/MUM1, but lacked pan-B markers such as CD79a, CD20, Pax-5, Bcl-2, and C-myc (5, 9). HHV-8, EBER, kappa simplex and CD45/LCA were also positive. Furthermore, the Ki-67 proliferation index exceeded 95%, findings completely consistent with the pattern of PEL (9). Given the absence of fluid accumulation in the abdomen, pericardium, pleura, or other body cavities, this case was classified as a rare extracavitary or solid variant of PEL (EC-PEL) (5).

Previous studies have demonstrated that 33 to 75% of patients with PEL also present with or have a history of KS (5). This observation implies that non-HIV-related PEL can serve as an ideal model for investigating the mechanisms underlying infection-to-tumor transformation under natural conditions, such as during physiological aging (8). The current case further corroborates this hypothesis. Notably, the progression from KS to PEL can occur not only between distinct organs (e.g., from skin to descending colon), but also within different regions of the same organ, such as the rectum, sigmoid colon, and descending colon. The characteristics of the masses evolve in accordance with morphological changes, exhibiting increased frequency, larger diameters, and firmer textures (Figure 1). Even within the same anatomical location, dynamic pathological features are evident. Pathological examination of the descending colon mass revealed typical B-cell phenotype lymphoma cells, slit-like angiogenesis, red blood cell accumulation, and a small number of atypical spindle cells expressing CD31 and CD34 (Figures 3D,E). These findings suggest that KS and EC-PEL may coexist within the same tissue (4). Additionally, a minor population of T cells expressing CD7 and CD3 (Figures 3K,M), as well as monocytes expressing CD163 (an inflammatory marker) (Figure 3L), were detected within the lesion. These observations may reflect the transitional state or intermediate phenotype of PEL during its progression from inflammation (such as KS) to malignancy (9, 16).

There is no doubt that KSHV is essential for KS to develop into PEL. The key is whether strain variation and *de novo* mutations occur in this process as with other DNA tumor viruses (17). Phylogenetic analysis based on the most variable gene ORF-K1 (VIP) suggested that KSHV DNA samples from blood (mtl A), saliva (mtl B), descending colon (mtl C), skin (mtl D) and gastric mucosa (mtl E) formed a single population with high support (ML/BI 88/0.92), which was attributed to genotype C. The DNA sequences of the five sites in this study were completely consistent without nucleotide differences, which was consistent with previous studies, that is, the lack of variability among multiple samples of the same patient (13). However, this is only for the highly variable VR1 domain of ORF-K1 gene, and subsequent sequencing of full-length K1 and more genes needs to be completed to verify whether KSHV has corresponding meaningful gene mutations in the process of inflammation to tumors. In addition to visible lesions in the skin and gastrointestinal tract, DNA sequences expressing ORF-K1 (VIP) gene were obtained in the blood and saliva of the patients, again demonstrating the key role of KSHV lytic infection in tumorigenesis (8), and the presence of viremia may be used as an indicator of the prognosis of patients.

Remarkably, our five samples differ in evolutionary position from the subtypes C1, C2, C3, and C7, and may be a new subtype, which we tentatively named Cnew (Figure 4). Although the correlation between the new genotype and the clinical phenotype (especially tumor progression) remains to be further investigated (12), the patient’s ethnic background of Bouyei nationality may be related to the new subtype. It is well established that the PCR fragment polymorphism correlate with geographic clustering of viral subtypes, reflecting patterns of human migration and ethnic divergence (4, 12, 18). Notably, KSHV exhibits male predominance (4), and its phylogenetic clustering demonstrates similarities to human Y-chromosome SNP patterns (19). Furthermore, the ancestry of nearly all modern Y chromosomes can be traced back to a common African origin (20). Thus, we hypothesize that KSHV infection in early human populations initiated long-term co-evolution with this virus. In-depth study of these viral genotypes could serve as valuable markers for revealing human evolution and migration patterns.

In summary, we have reexamined this easily overlooked virus, KSHV, through an in-depth study of the first global case of concurrent KS and PEL in Bouyei nationality. As an ancient virus that coevolved with humans, from an epidemiological point of view, why does it have a much lower penetrance than EBV, and did it acquire immune escape ability at some stage of its life cycle? From the perspective of immunology, since it coexists with humans, does it have both disadvantages and advantages for humans? From a clinical perspective, how does KSHV dynamically regulate the development of inflammation to tumor spectrum disorders? Since it acts on vascular endothelial cells to promote vascular proliferation, is KSHV associated with other infectious or immune diseases? Addressing these questions is imperative to unravel its mysteries.

## Data Availability

The raw data supporting the conclusions of this article will be made available by the authors, without undue reservation.

## Supplementary material

SUPPLEMENTARY FIGURE S1 Multiple biopsies of the antrum, body, ileum and rectum showed chronic inflammation, with intervening slit-like spaces and red blood cell extravasation **(A–D)**.

SUPPLEMENTARY FIGURE S2 Timeline of symptoms and diagnosis.
