# Supplementary figures and images for: Case Report: From Kaposi’s sarcoma to primary effusive lymphoma
Source: Front Med (Lausanne). 2025 Aug 12;12:1591462. doi: 10.3389/fmed.2025.1591462 (PMC12379098; doi:10.3389/fmed.2025.1591462)

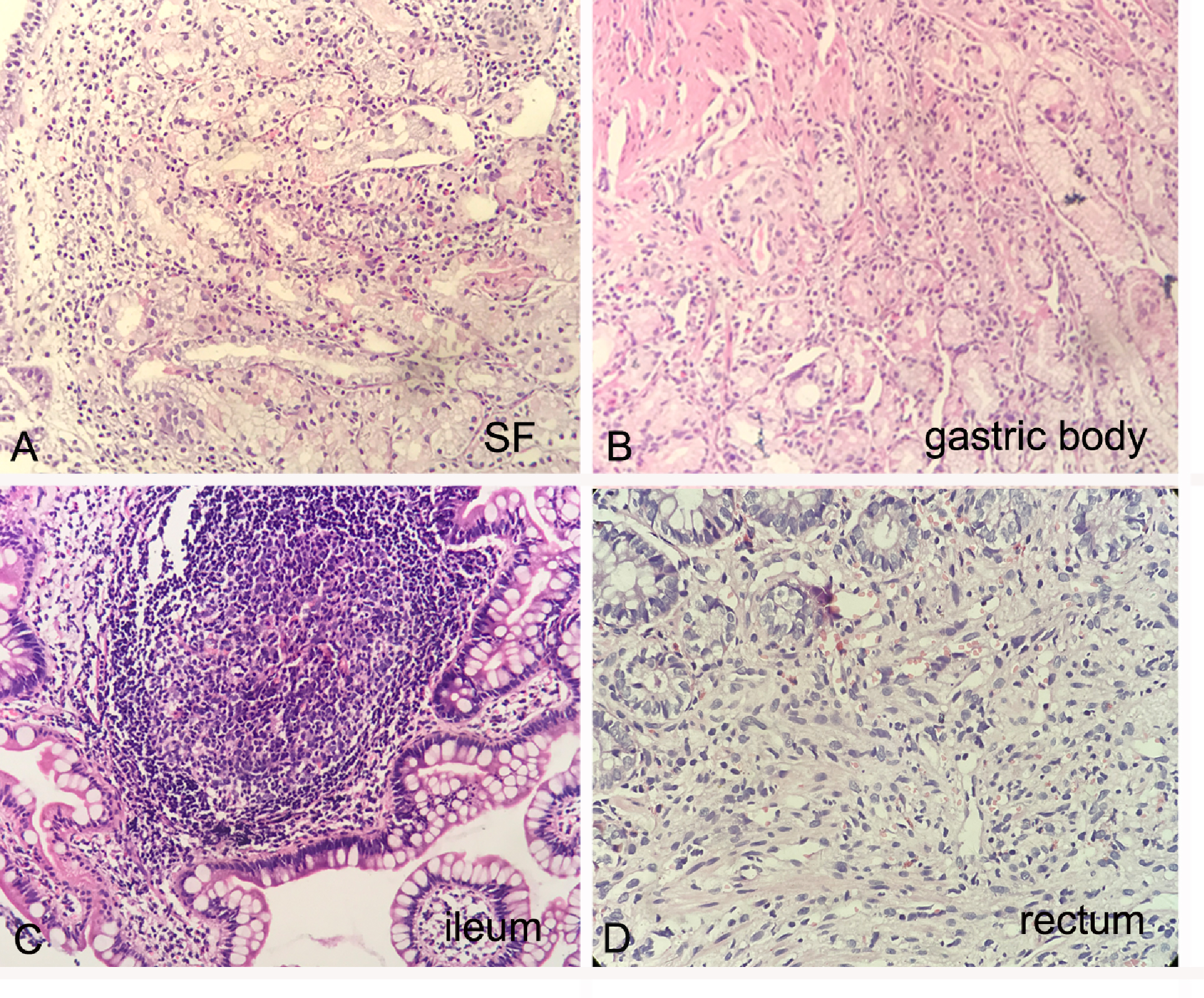

Supplement: SUPPLEMENTARY FIGURE S1 — Multiple biopsies of the antrum, body, ileum and rectum showed chronic inflammation, with intervening slit-like spaces and red blood cell extravasation (A–D). [file Image_1.JPEG]

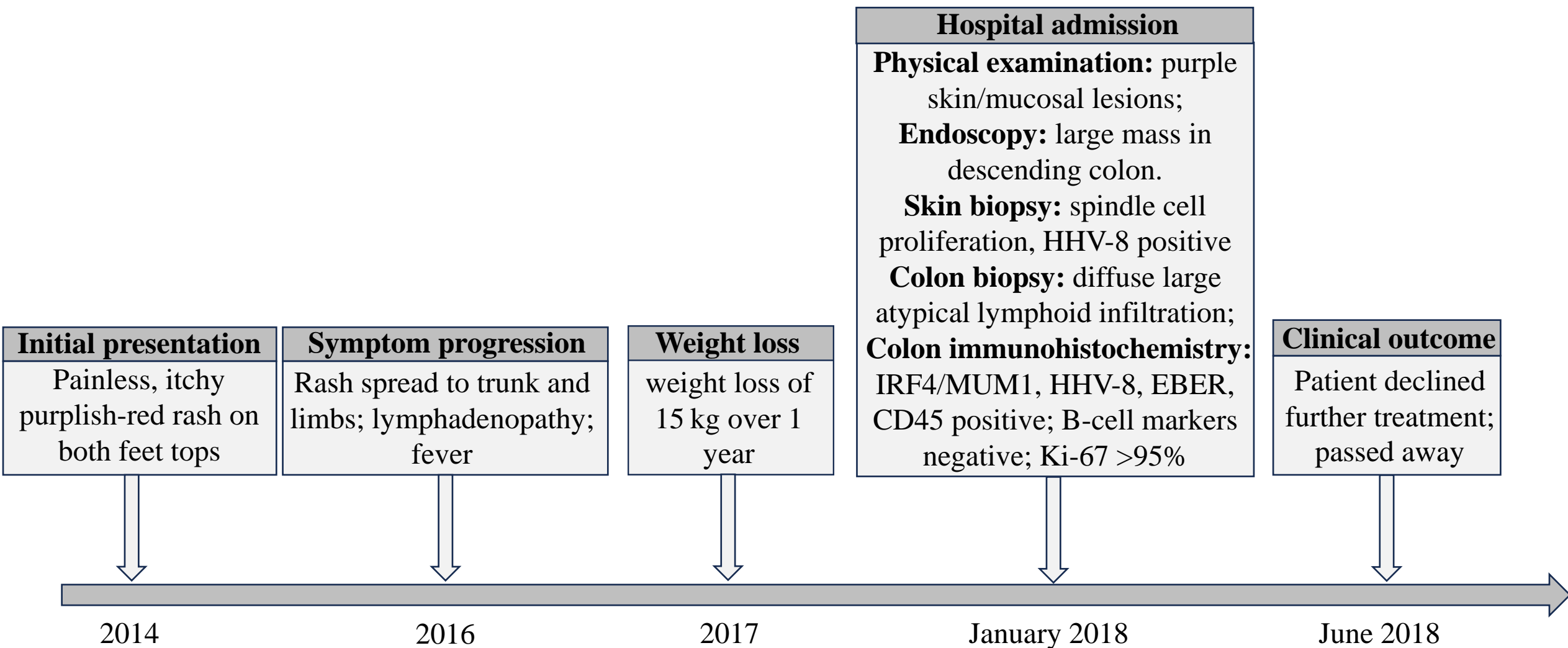

Supplement: SUPPLEMENTARY FIGURE S2 — Timeline of symptoms and diagnosis. [file Image_2.pdf]
